# Supplementary material for: Preclinical Head and Neck Squamous Cell Carcinoma Models for Combined Targeted Therapy Approaches
Source: Cancers (Basel). 2022 May 18;14(10):2484. doi: 10.3390/cancers14102484 (PMC9139292; doi:10.3390/cancers14102484)
Supplement: Supplementary file 1 [file cancers-14-02484-s001.zip › cancers-1719893-supplementary.pdf]

cancers

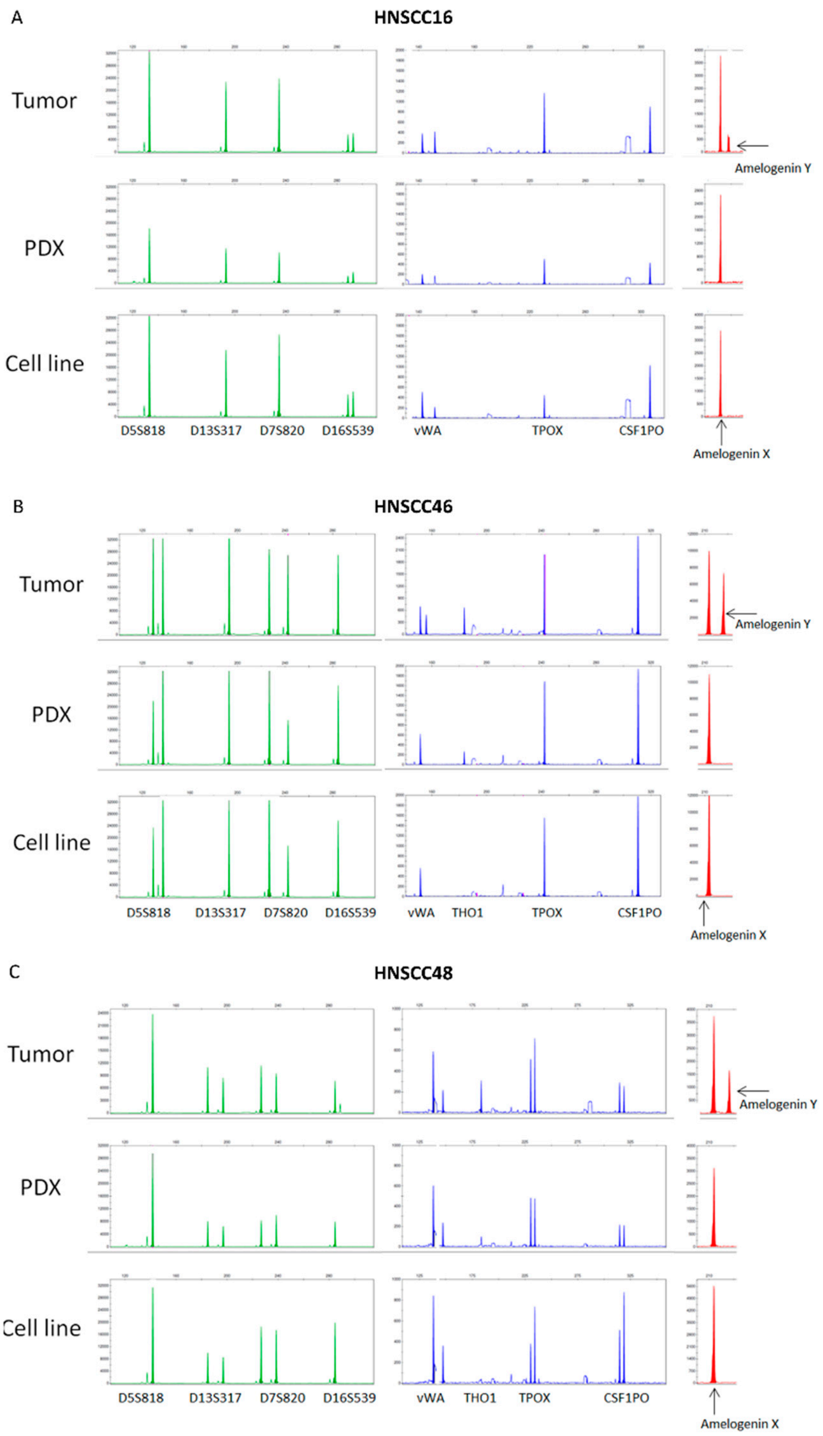

Figure S1. Fingerprint PCR of patients' tumor, PDX and cell line of (**A**) HNSCC16 (**B**) HNSCC46 and (**C**) HNSCC48 to confirm identity with the patient. Used are the following loci: D5S818, D13S317, D7S820, D16S539, vWA THO1, TPOX, CSF1PO and Amelogenin X and Y. HEX (green), FAM (blue), TAMRA (red).

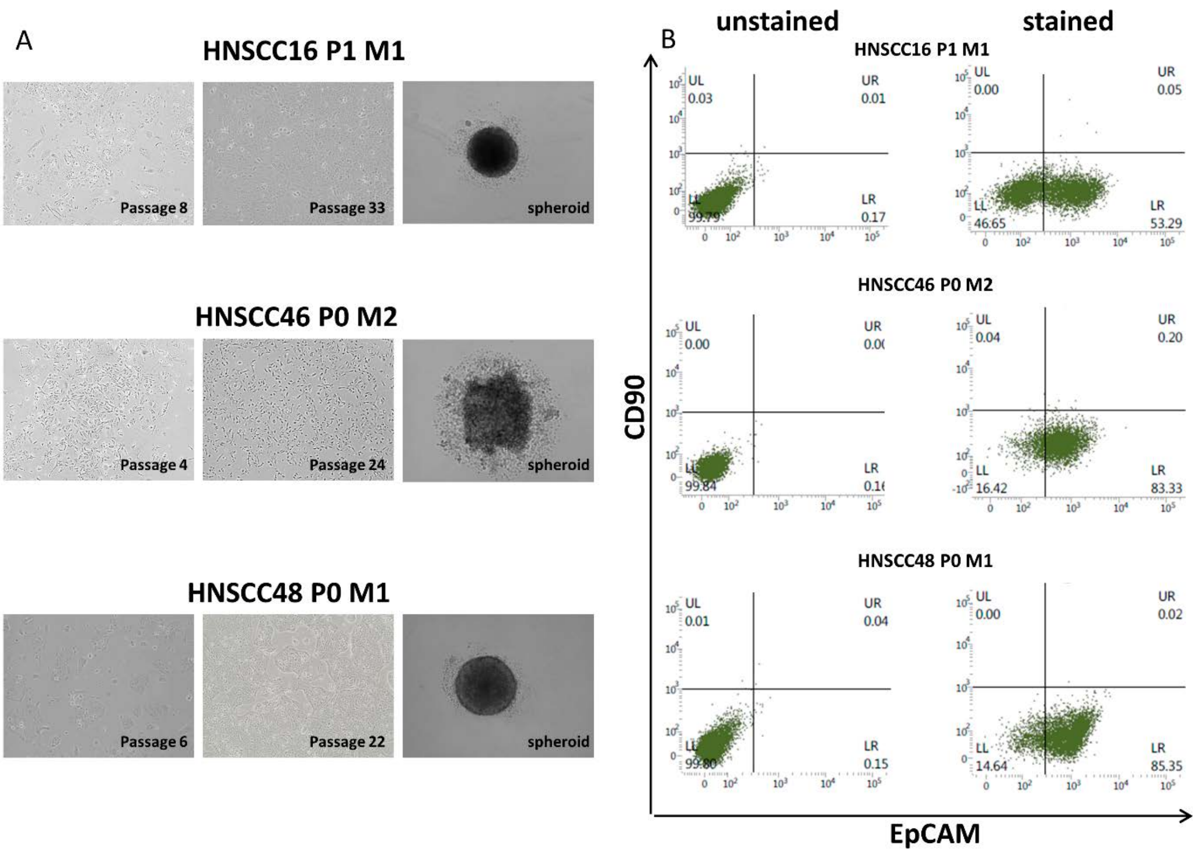

Figure S2. Establishment of xenograft-derived cell lines. (**A**) Light microscopy images of HNSCC16 P1 M1, HNSCC46 P0 M2, and HNSCC48 P0 M1 in early passages(passage 4-8), late passages (passage 22-33), and spheroid formation after 5 days in culture. Original magnification 100x. (**B**) FACS analysis confirms absence of fibroblasts. Cells were stained with CD90 (fibroblast marker) and EpCAM (CD326, epithelial tissue marker).

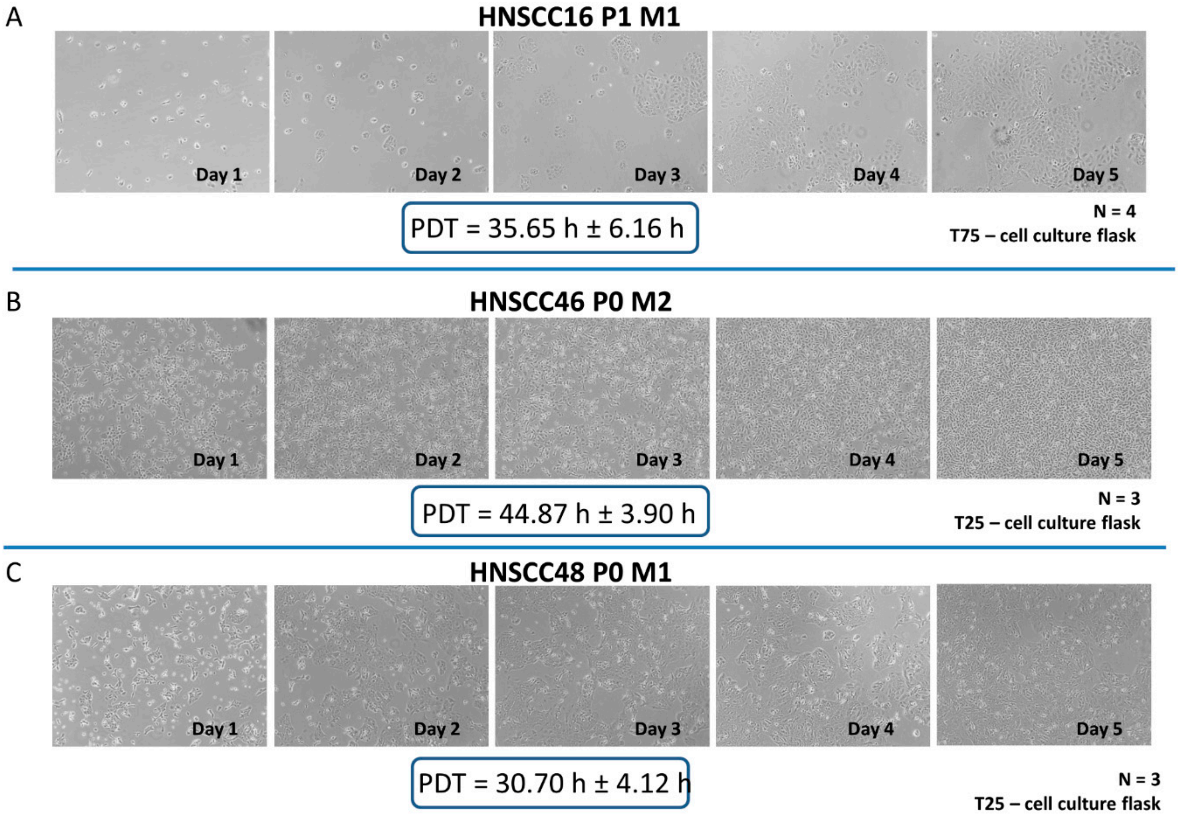

Figure S3. Population doubling times of (**A**) HNSCC16 M1 P1, (**B**) HNSCC46 P0 M2, and (**C**) HNSCC48 P0 M1. Shown are representative light microscopy images of the cells on 5 consecutive days after seeding a defined amount of cells. Original magnification 100x.

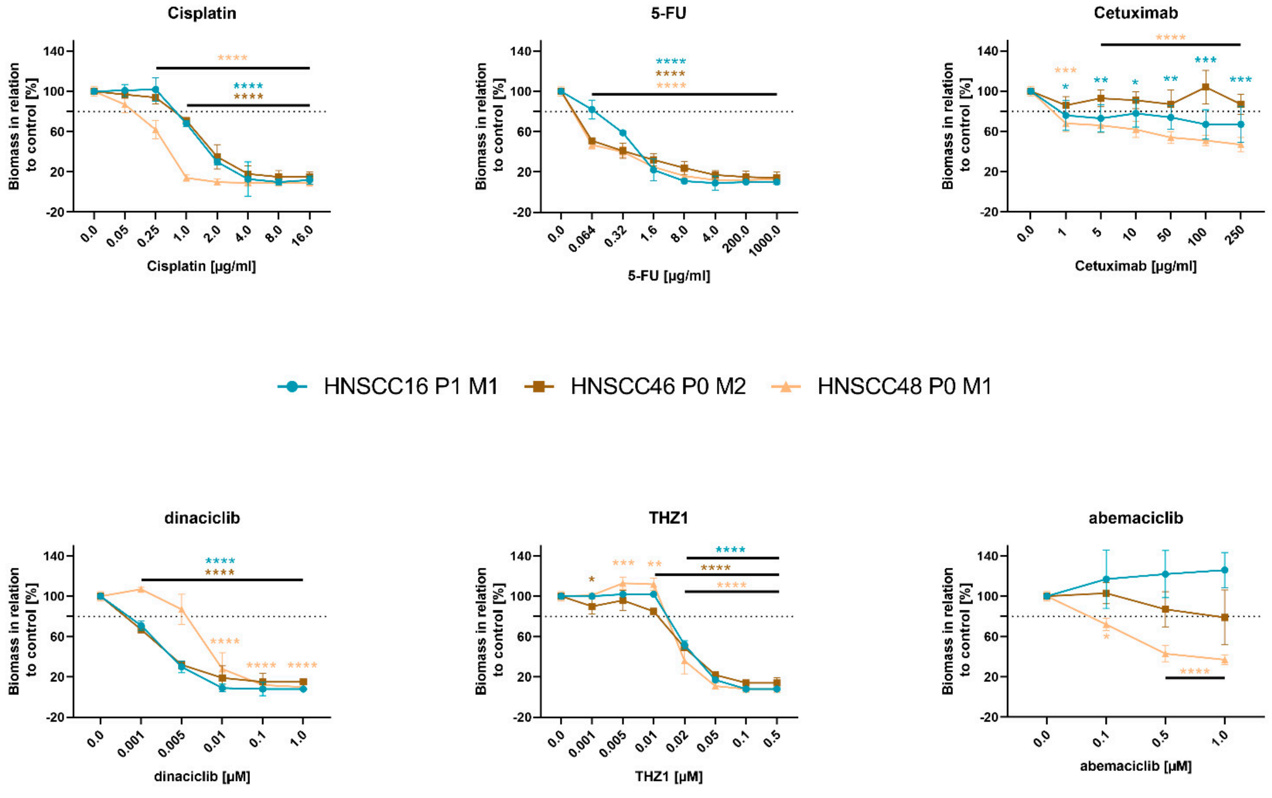

Figure S4. Dose–response curve analysis. Tumor cells were treated for 2 × 72 h with the different test substances in varying concentrations ranging between 0.048 µg/mL–1 mg/mL for approved drugs (Cisplatin, 5-FU, and Cetuximab) and 1 nM–1 µM for targeted substances (dinaciclib, abemaciclib, and THZ1). Based on the dose–response curves IC_{50} was calculated as outlined in Table 3. * $p < 0.05$; ** $p < 0.01$; *** $p < 0.001$; **** $p < 0.0001$ vs. control (100%). Two-way ANOVA.
